# Recurrent acute hemorrhagic necrotizing encephalopathy associated with RAN-binding protein-2 gene mutation in a pediatric patient

**DOI:** 10.1259/bjrcr.20220019

**Published:** 2022-11-01

**Authors:** Olutayo Ibukunolu Olubiyi, Carlos Zamora, Valerie Jewells, Senyene E. Hunter

**Affiliations:** 1 Division of Neuroradiology, Department of Radiology, University of North Carolina, Chapel Hill, North Carolina, USA; 2 Division of Pediatric Neurology, Department of Neurology, University of North Carolina, Chapel Hill, North Carolina, USA

## Abstract

A young male child presented with recurrent episodes of seizures and altered mental status following febrile episodes on three separate occasions between his first and third birthdays. Laboratory evaluations identified SARS-CoV-2 infection during the first episode and no infective agents or antibodies in the cerebrospinal fluid during all the episodes. Brain imaging with CT and MRI revealed bilaterally symmetric patchy hemorrhagic necrotic foci in the deep brain nuclei and medial temporal lobes, prompting suspicion for an underlying predisposition to recurrent acute hemorrhagic necrotizing encephalopathy. Gene analysis confirmed a mutation in the RAN-binding protein-2 (RANBP2) gene. The patient made good recovery following treatment with IVIG, steroids and plasmapheresis, and follow-up brain imaging showed no progression of brain lesions.

Early suspicion from characteristic imaging features in appropriate clinical settings will inform timely appropriate treatment and better outcome. We therefore provided short review of imaging features of acute hemorrhagic necrotizing encephalopathy.

## First presentation

An otherwise healthy 2-year old male with prior history of seizures and respiratory failure necessitating mechanical ventilation following a febrile episode, presented to the emergency department with fever, progressive lethargy, and respiratory failure requiring intubation.

### Investigations

A nasal swab test was positive for SARS-CoV-2 with clinical and laboratory findings consistent with multisystem inflammatory syndrome. Cerebrospinal fluid (CSF) analysis showed pleocytosis and a white blood cell count of 16 /mm^3^. CSF was unremarkable for bacterial agents or viral PCR (adenovirus, enterovirus, HSV1 and 2), and serum was negative for SARS-CoV-2 IgG.

**Figure 1.**
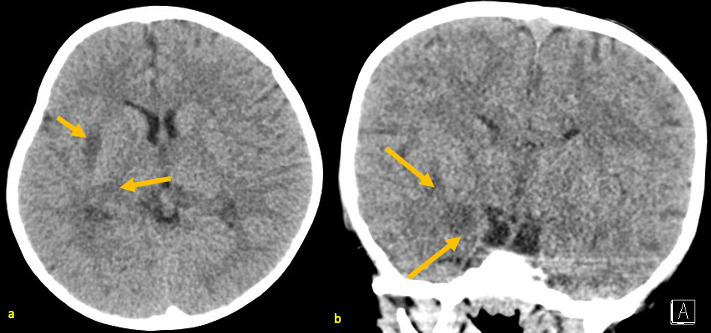
A 25-month-old male with AHNE. Non-contrast head CT obtained during his first presentation for AHNE: axial (**a**) and coronal (**b**) views showing patchy hypodensities in the right external capsule, right posterior internal capsule, and right medial temporal lobe. These were non-specific and were later correlated to areas of acute necrosis on subsequent brain MRI. AHNE, acute hemorrhagic necrotizing encephalopathy.

**Figure 2.**
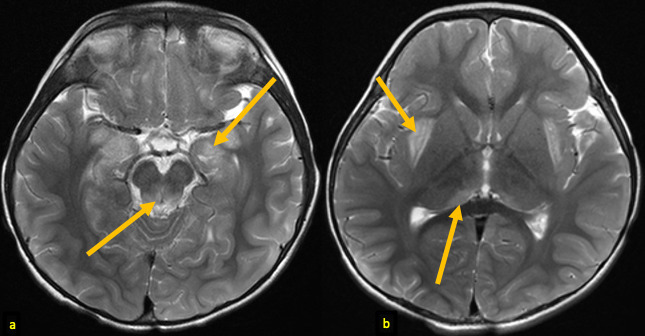
A 25-month-old male with AHNE. Initial brain MRI obtained during his first presentation for AHNE: axial *T*_2_ weighted images (**a, b**) demonstrating hyperintense signal abnormalities (arrows) in medial temporal lobes, periaqueductal regions, posteromedial thalami, and subinsular regions. Note the bilateral and relatively symmetric distribution of the lesions. AHNE, acute hemorrhagic necrotizing encephalopathy.

**Figure 3.**
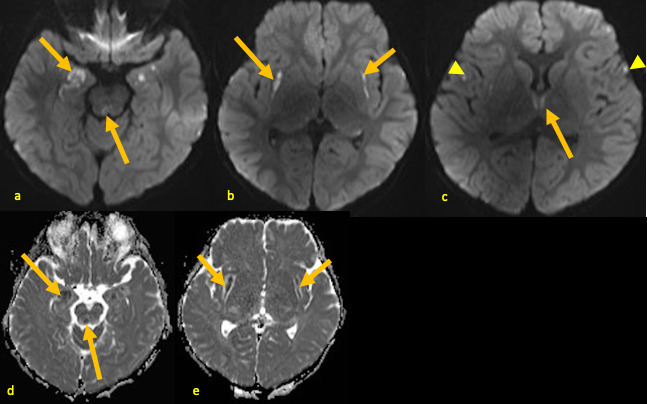
A 25-month-old male with AHNE. Brain MRI obtained during his first presentation for AHNE: axial DWI (**a–c**) and ADC maps (**d, e**) demonstrating patchy (arrows) hyperintense DWI signal abnormalities and corresponding low ADC values in medial temporal lobes, periaqueductal regions, medial thalami, and subinsular areas consistent with acute necrosis. Additional punctate foci of diffusion restriction are also noted in frontal cortices (arrowheads), likely postictal changes. ADC, apparent diffusion coefficient; AHNE, acute hemorrhagic necrotizing encephalopathy; DWI, diffusion-weighted image.

**Figure 4.**
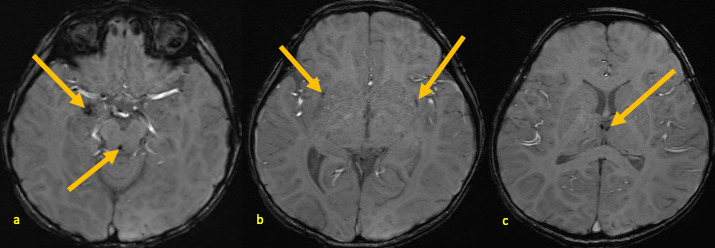
A 25-month-old male with AHNE. Brain MRI obtained during his first presentation for AHNE: SWI (**a–c**) demonstrating foci of low signal abnormality consistent with areas of microhemorrhages (arrows) in the right medial temporal lobe, periaqueductal regions, bilateral subinsular areas, and bilateral medial thalami. AHNE, acute hemorrhagic necrotizing encephalopathy; SWI, susceptibility-weighted image.

Non-contrast head CT [Figure 1] demonstrated decreased attenuation in the right temporal lobe, subinsular region and external capsule. Further imaging with MRI of the brain [Figures 2–4] demonstrated multifocal signal abnormalities with associated foci of restricted diffusion and microhemorrhages involving the medial temporal lobes, subinsular areas, and thalami bilaterally, concerning for acute hemorrhagic necrotizing encephalopathy (AHNE). Additional foci of abnormal T2/fluid attenuation inversion recovery (FLAIR) signal were noted in the subinsular and periaqueductal regions.

### Differential diagnosis

Acute encephalitis or meningitis were other top differential diagnostic considerations. The patient was ultimately diagnosed with AHNE related to SARS-CoV-2 infection based on the imaging findings and laboratory results.

### Treatment

He was treated with intravenous immune globulin (IVIG) and dexamethasone leading to rapid clinical improvement. Shortly thereafter, the patient was weaned off mechanical ventilation, extubated, and discharged.

## Second presentation

The patient presented again 5 months later with status epilepticus and fever of 102.4°F.

### Investigations

CSF analysis demonstrated markedly elevated IL-6 (1596 pg ml^−1^) and IL-8 (3225 pg ml^−1^) with minimally elevated IL-2, IL-4, and IL-10, consistent with an inflammatory process.

**Figure 5.**
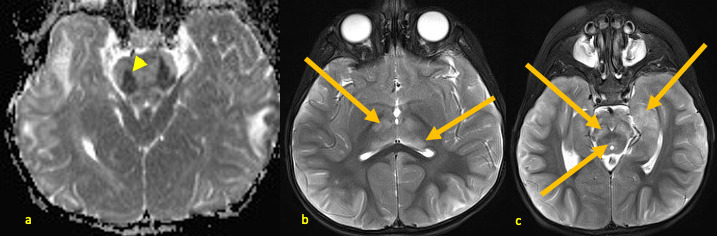
Now 30-month-old male with rAHNE. Brain MRI obtained during his second presentation for AHNE: axial ADC map (**a**) demonstrating low signal (arrowhead) in the pons; axial *T*_2_ weighted images (**b, c**) demonstrating hyperintense signal abnormalities (arrows) in bilateral anterior and posteromedial thalami (**b**), medial temporal lobes, midbrain, & periaqueductal regions (**c**). ADC, apparent diffusion coefficient; rAHNE, recurrent acute hemorrhagic necrotizing encephalopathy.

**Figure 6.**
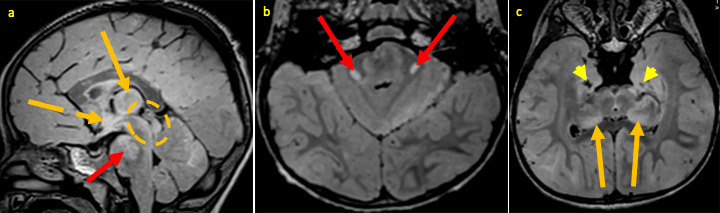
30-month-old male with rAHNE. Brain MRI obtained during his second presentation for AHNE: sagittal (**a**) and axial (**a, c**) FLAIR images demonstrating new hyperintense signal abnormalities in the subthalamic area [broken orange arrow, (**a**)], pons extending into middle cerebellar peduncles [red arrows (a & b)], and midbrain [broken orange circle (**a**)] as well as worsening hyperintense signal abnormalities (orange arrows) in the thalami (a & c). Note areas of evolving encephalomalacia (yellow arrow heads) in bilateral medial temporal lobes (**c**). AHNE, acute hemorrhagic necrotizing encephalopathy; FLAIR, fluid attenuation inversion recovery.

Brain MRI demonstrated new and worsening patchy signal abnormalities on *T*_2_ weighted and FLAIR sequences with new areas of acute necrosis on diffusion-weighted images (DWIs) [Figures 5 and 6] and foci of microhemorrhage on susceptibility-weighted images (SWIs), predominantly involving the midbrain, pons, and middle cerebellar peduncles. These were consistent with a new episode of AHNE and the patient was diagnosed with recurrent AHNE (rAHNE). This raised a suspicion for an underlying predisposition for rAHNE prompting further evaluation with genetic analysis. Genetic analysis confirmed RAN-binding protein-2 gene mutation.

### Treatment

The patient was treated with corticosteroids, IVIG, tocilizumab, and plasma exchange.

### Outcome and follow-up

**Figure 7.**
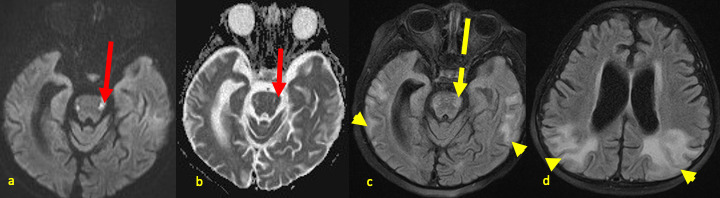
Now 35-month-old male with rAHNE. Follow-up brain MRI obtained 18 weeks after the last acute episode displayed in Figure 3, axial images demonstrating evolving sequelae of hemorrhagic necrotizing encephalopathy: DWI (**a**) & ADC (**b**) showing bilateral signal abnormalities in lateral pons (red arrows); FLAIR images (**c, d**) showing new cortical/subcortical hyperintense signal abnormality in the temporal and parietal lobes (arrow heads), with decreasing pontine signal abnormality (broken arrows). Note the worsening diffuse parenchymal volume loss with ex-vacuo dilatation of lateral ventricles and widening of sulci. ADC, apparent diffusion coefficient; DWI, diffusion-weighted image; FLAIR, fluid attenuation inversion recovery; rAHNE, recurrent acute hemorrhagic necrotizing encephalopathy.

Patient’s fever resolved, his seizures were controlled, and he was discharged to acute inpatient rehabilitation then home to outpatient follow-up. Patient subsequently manifested developmental delay. Follow-up brain MRI performed 10 weeks after discharge from the hospital revealed evolving changes from prior brain lesions, marked diffuse cerebral volume loss, and new cortical/subcortical signal abnormalities in the temporal and parietal lobes [Figure 7].

## Discussion

AHNE is a rapidly progressing encephalopathy that most commonly occurs in the setting of viral infection.^1–3^ Typically, AHNE follows an initial febrile episode with rapid progression to altered mentation with or without seizures, such as in the case presented here.^2,3^ There may also be varying degrees of flaccid or spastic paralysis, bladder or fecal incontinence, and speech disability. Commonly implicated viral agents include SARS-CoV-2 (COVID-19) virus, influenza A and B, adenovirus, enterovirus, human herpes virus 6 and 7, rubella, and measles.^4–6^ Mycoplasma is a non-viral agent that has also been implicated in AHNE.^5^

On brain imaging, AHNE typically presents with bilateral and symmetric multifocal lesions. Key areas of involvement include the thalami, brainstem, and cerebral white matter, while the tegmentum, cerebellum, putamen and internal capsule may also be involved.^5^ CT has low sensitivity but may show hypoattenuation in affected areas corresponding to edema or foci of increased attenuation due to hemorrhage.^4^ On MRI, involved areas appear edematous exhibiting hypointense T1-signal and hyperintense T2/FLAIR signal, with variable diffusion restriction manifesting as hyperintense DWI signal with corresponding dark signal on ADC maps. Varying amounts of hemorrhage, more commonly microhemorrhages present as dark foci of blooming artifact on SWI. However, frank parenchymal hematomas have been known to occur. Some lesions may exhibit peripheral contrast enhancement.^4,5,7^ Cavitation develops within the affected areas, but typically not in the acute phase. These imaging features are exemplified in Figures 1–4 above. Note that the previously described “classical neuroimaging” feature of DWI tricolor pattern or target-like lesions best seen on ACD maps is typically seen with larger lesions^8^ and was not present in our case.

In recurrent acute necrotizing encephalopathy, MRI demonstrates acute lesions that may show a different distribution superimposed on areas of subacute and/or chronic lesions from prior episodes [Table 1]. The latter typically manifests as patchy areas of encephalomalacia in typical locations, and varying degrees of parenchymal atrophy.^9^ In the present case, differences in the pattern of signal abnormalities between the studies may have been related to evolution of the initial lesions and/or areas of new injury in the setting of recurrent encephalitis. Presumably, some of the new cortical changes, particularly in the temporal lobes, could have also represented sequelae of prolonged status epilepticus during the acute episode. Similarly, prior studies have shown that MRI findings may with clinical outcomes in confirmed cases.^5^ Wong et al calculated imaging scores for 12 patients based on the presence of hemorrhage, cavitation, and location of lesions, and correlated this to their clinical outcome categories based on health state utility value.^5^ This yielded a strong positive correlation (*r* = 0.76, *p* = .001) between the MRI score and the outcome category. Note that the presence of hemorrhage in acute necrotizing encephalopathy is a recognized independent predictor of poor outcome, therefore the importance of identifying this feature and properly categorizing the disease as AHNE.

**Table 1.** Summary

	**Recurrent acute hemorrhagic necrotizing encephalopathy**
Etiology	Triggered by a febrile viral infection in a person with genetic susceptibility such as RANBP2 gene mutation (or less frequently, other similar gene mutation).
**Incidence**	Very rare; estimated as 20% of people with the mutated RANBP2 gene allele (due to incomplete penetrance).
**Gender ratio**	1:1
**Age predilection**	Peaks at age 6–18 months.
**Risk factors**	Underlying heterozygosity or homozygosity for RANBP2 gene mutation, with a febrile viral infection
**Treatment**	IVIG, plasmapheresis, and steroid
**Prognosis**	30–40% mortality, and 15–35% severe permanent neurologic deficit in survivors.
**Findings on imaging**	Mixture of acute, subacute, and chronic lesions Acute: patchy T2/FLAIR hyperintensity with diffusion restriction, blooming hypointense SWI foci/areas of micro-/macrohemorrhage. Chronic: foci/areas of encephalomalacia, diffuse atrophy, SWI blooming foci may persist.
**Preferentially involved regions**	Bilaterally symmetric in the thalami, putamen, internal capsules, subinsular region, periaqueductal gray, pons

FLAIR, fluid attenuation inversion recovery;IVIG, intravenous immunoglobulin; RANBP2, RAN-binding protein-2.

A missense mutation in the RANBP2 gene located on chromosome 2q which encodes the nuclear pore component Ran binding protein 2, is a genetic risk factor for an environmentally triggered acute necrotizing encephalopathy.^3^ The mutation produces an autosomal dominant allele that exhibits variable penetrance. Although the pathogenesis from the nuclear protein to AHNE is not fully understood, the leading hypothesis is that a “cytokine storm” is the cardinal process.^3^ This idea stems from the findings of elevated serum and CSF levels of IL-6 and TNF-α identified in most patients afflicted by the disease.^3,6^ A cytokine storm refers to aberrant and unregulated production of soluble chemical immune mediators (cytokines and chemokines) and the accompanying immunopathology.^10^

Although a cytokine storm can have systemic implications affecting multiple organs, prior studies have shown that in patients with RANBP2 gene mutation, the lesions of ANE1 are largely localized to the CNS, especially affecting the deep gray matter of the brain.^4,5,7,8^ A missense mutation in the gene encoding this protein produces a loss of function leading to a positive feedback loop during acute stress periods that ultimately culminates in a cytokine storm via the TNF and IL-6 pathways.^11–13^ Similarly, cytokine storm-mediated tissue injuries have been reported in the extracranial CNS in both ANE1 and non-familiar cases of ANE, presenting as multifocal or longitudinally extensive myelitis.^14–16^

It should be noted that several disease entities can mimic AHNE clinically and even radiologically. However, attention to the cardinal imaging features of AHNE and the clinical setting can ensure an appropriate diagnosis in most cases. For example, herpes encephalitis commonly mimics AHNE presenting with a febrile prodrome followed by rapid cognitive deterioration. Herpes encephalitis may also lead to hemorrhagic foci in the medial temporal lobes, but typically spares the thalami and brain stem, allowing for differentiation.^5^ Furthermore, CSF analysis in HSV encephalitis follows an encephalitic pattern with pleocytosis and elevated proteins in addition to probable presence of the viral antigen. The rare dengue virus encephalitis may closely mimic AHNE by involving the thalami, basal ganglia, and pons bilaterally with a tendency for petechial hemorrhage. However, imaging findings are variable.^17^ In addition to prominent cortical involvement of the temporal lobes, dengue virus encephalitis almost always coexists with multiorgan involvement, which may be hemorrhagic, unlike AHNE.^17^ Several other viral encephalitides may involve the central gray matter, brainstem, and cortex to varying degrees.

Similarly, in many of the metabolic encephalopathies such as hemolytic uremic syndrome, toxic encephalopathy, hemorrhagic shock, and encephalopathy syndromes, brain lesions are less frequently bilateral and symmetrical.^5^ Although hypoxic encephalopathy often affects the deep gray matter and can be symmetric in distribution, it frequently affects both basal ganglia and cortex unlike AHNE.^5^ History of a preceding event with profound hypoxia is often present. Vasculitides are a heterogeneous group of conditions with varying distribution on imaging depending on the type of vessels involved—large *vs* medium or small vessels, or mixed type.^18^ However, vasculitides usually produce multifocal asymmetric infarcts, often involving multiple bilateral vascular territories without a deep gray matter predilection unlike AHNE.^18^ Vasculitides can also be complicated by hemorrhage. When involving large vessels, lesions typically occur along the area of distribution of the affected vessels and may be recurrent like AHNE. As opposed to AHNE, angiography may be positive in vasculitis although the overall sensitivity is low.^18^

## Teaching point

- Bilaterally symmetric hemorrhagic necrosis predominantly involving the thalami, brainstem, and/or cerebral white matter in a pediatric patient with fever and neurocognitive symptoms should raise suspicion for AHNE.
- Prior history or recurrence of similar episodes, or imaging findings of old necrotic foci at typical deep brain locations should raise suspicion for recurrent AHNE with possible underlying predisposition, and prompt genetic analysis for conditions like RANBP2 gene mutation.
